# A Prospective Study of Sutured Versus Non-sutured Subcutaneous Fat Tissue in Laparotomy Wound Closure

**DOI:** 10.7759/cureus.62034

**Published:** 2024-06-09

**Authors:** Jasira Padinhare Madathil, Revathy P Kumar, T. V. Haridas, Jim Job, Pradeep Chandran, Jayas Siby

**Affiliations:** 1 Breast and General Surgery, Homerton University Hospital, London, GBR; 2 Plastic Surgery, Government Medical College, Kozhikode, Kozhikode, IND; 3 General Surgery, Government Medical College, Thrissur, Thrissur, IND; 4 Upper Gastrointestinal and Bariatric Surgery, Princess Royal University Hospital, London, GBR; 5 Trauma Surgery, King's College Hospital, London, GBR; 6 General Surgery, Whipps Cross University Hospital, London, GBR; 7 General Surgery, Royal Sussex County Hospital, Brighton, GBR

**Keywords:** surgical wound complications, wound closure, laparotomy, sutured, subcutaneous fat

## Abstract

Background

This study was conducted to determine the wound-related complications, such as wound dehiscence, delayed post-operative stay, and reinterventions in both groups, and compare the incidence of surgical site infection in elective laparotomy wounds in two groups - those with closing subcutaneous fat tissue and those without.

Methods

At the Government Medical College, Thrissur, 248 patients undergoing elective abdominal surgeries during the period from August 2019 to August 2020 participated in this one-year prospective cross-sectional study. The institutional ethics committee approved the study, and participants provided written informed consent. Patients were randomly assigned to the subcutaneous closure group (S) and the non-closure group (N). Post-operative events were then systematically documented.

Results

The group with no subcutaneous suture (N) had a considerably greater percentage of patients with seromas (12 patients, 9%), hematomas (13 patients, 10.5%), superficial surgical site infection and total wound dehiscence as compared to the subcutaneous tissue closure group (S). Groups N and S displayed comparable numbers of suture sinus developments and partial wound dehiscence. Group N and Group S had similar hospital stays, according to the overall length of stay displayed.

Conclusion

Subcutaneous suturing during elective laparotomy wound closure significantly reduced superficial surgical site infection, hematoma, seroma, and total wound dehiscence; in the remaining categories, subcutaneous and non-subcutaneous sutures did not differ significantly. It also did not help to reduce hospital stays.

## Introduction

Many surgical procedures involve abdominal surgical access [1]. From the inside out, the layers that make up the anterior abdominal wall are the peritoneum, muscle, superficial fascia, subcutaneous tissue, and skin. One topic that is still explored in abdominal surgery is the suturing of subcutaneous fat tissue during surgical wound closure. There is debate concerning suturing subcutaneous tissue following surgery. While some surgeons believe it is unneeded and could worsen wound issues, others advise its use, arguing that it reduces wound complications. Because the tissue is kept close together until sufficient healing has taken place to resist tension without the need for artificial support; suture closure facilitates initial wound healing. A foreign body tissue reaction is caused by suture material, which is inserted in human tissue. To reduce the risk of wound infection during wound closure, a sterile area and careful aseptic procedures are essential. Hypertrophic scars, broad scars, and wound dehiscence are examples of wound healing complications that can be brought on by patient factors such as poor nutritional status, improper suture selection, or a technique that applies too much stress throughout the wound [2]. Of the estimated 27 million surgical procedures performed annually, 500,000 or so result in surgical site infections (SSIs) [3].

A number of factors, such as the type of surgery, the wound closure technique used, the patient's underlying systemic illnesses, the medications they are taking (including antibiotics for infections and other drugs that may impair wound healing), the antiseptic measures taken to prevent wound complications, and the duration of follow-up, all affect the incidence of wound healing complications, such as SSI and wound dehiscence (wound breakdown along the incision) [4]. In surgical patients, post-operative SSIs continue to be a significant cause of morbidity and a less common cause of death. Because SSI can have a wide range of clinical manifestations, it is a challenging phrase to describe precisely. The Centers for Disease Control and Prevention (CDC) defines SSI as an enlargement of pathogenic microorganisms that grow in an incision site, either in the muscle-fascial layers (deep), the skin and subcutaneous fat (superficial), or, if an organ or cavity is opened during surgery, in the organ [5]. A skin surface infection must be diagnosed in conjunction with clinical signs such as redness, heat, pain, swelling, separation of the suture line (dehiscence), or the presence of an abscess in the deeper tissues. This is because the skin is normally colonized by bacterial flora. Patients may develop sepsis symptoms or SIRS (systemic inflammatory response syndrome), which would increase morbidity and death [6]. SSIs are the biological culmination of several compounding factors, such as the patient's host defence mechanisms, the specific virulence of contaminants, the microenvironment of each wound, and the inoculum of bacteria introduced into the wound during the procedure [7]. Even though a zero SSI rate may not be possible, ongoing research into the biology of SSIs and regular use of tried-and-true preventative techniques may enable further reductions in the incidence, expense, and morbidity of SSI. Superficial incisional SSI, deep incisional SSI, and organ/space SSI are the three sub-classifications of SSIs. Infections at the surgical site following surgery can lengthen hospital stays and raise medical expenses. Poor cosmesis can result from either hypertrophic scarring or keloid scarring brought on by impaired wound healing. A lower quality of life might also arise from poor wound healing. The understanding that suturing delicate fat tissue is problematic and that the suture material exacerbates generally inadequate fat perfusion and raises the risk of surgical complications is the basis for opposition to suturing the fat layer. Moreover, sutures could serve as a focal point for bacterial contamination and wound infection because they are an external material. The fat layer is segmented by stitches, which could lead to several distinct fluid deposits. Subcutaneous suturing is typically used on obese patients to better match the skin edges; nevertheless, this suturing is never flawless, and tissue disintegration may result from ischemia. The purpose of this research is to compare the rate of SSI in two groups of elective laparotomy wounds - one with closed subcutaneous fat tissue and the other without - as well as to identify additional complications related to the wound, such as dehiscence, delayed recovery from surgery, and repeat interventions in both groups.

## Materials and methods

This is a prospective cross-sectional study. A total of 248 patients who underwent elective abdominal surgeries from August 2019 to August 2020 at the Government Medical College, Thrissur were the study population. The Institutional Ethics Committee approved the study, and participants were provided with written informed consent. All patients who underwent elective laparotomies in all units of the department of general surgery, irrespective of indication for surgery, comorbidities and other perioperative factors were included in the study. Those who had emergency abdominal surgeries, contaminated wounds and those who were not willing to participate were excluded. The sample size was calculated based on a study comparing subcutaneous closure versus no subcutaneous closure after a non-caesarean surgical procedure, a systematic review conducted by Gurusamy et al. (2014) [8]. The total estimated size was 248 patients. Hence, 124 patients were included in each group. The subcutaneous closure group were represented as the S group and the non-subcutaneous closure group as N.

Patients were randomly assigned to the subcutaneous closure group (S) and non-subcutaneous closure group (N). Computer-generated random sampling methods were used. In the non-subcutaneous tissue closure group (N), the abdomen was closed in two layers: linea alba with No. 1 polydioxanone loop and skin with skin staplers. In the subcutaneous closure group (S), along with these two layers, the middle subcutaneous tissue was closed with 3-0 polyglactone (Vicryl; Ethicon, Inc., Somerville, USA). The wounds were then inspected for evidence of wound-related complications until they left the hospital and they were followed up until after 30 days from surgery by the principal investigator. Patients attended routine weekly outpatient reviews and attended emergency if any worrying symptoms developed. All the follow-ups were properly documented with a general examination and examination of the wound for any complications. In case of any suspected infection, a wound swab was sent to microbiology for culture and sensitivity. Other reported wound-related complications such as total wound dehiscence, partial wound dehiscence, seroma, hematoma and suture sinus formation were also documented and compared. The total length of hospital stay post-operatively was also documented and analyzed.

Statistical Package for the Social Sciences (SPSS Statistics for Windows, Version 17.0. Chicago: SPSS Inc.) was used to tabulate and analyze all of the findings, which were entered into an Excel sheet (Microsoft® Corp., Redmond, WA, USA). A student t-test was used to compare two arithmetic means. Two proportions, or percentages, were compared using the Chi-square test. The threshold for statistical significance (p) was set at 5%.

There are no major ethical concerns with the study as both groups of patients were taken up for standardized procedures currently in practice. No interventions that could possibly change the outcome of their disease were used.

## Results

In the study, there were a total of 248 patients randomly assigned to the subcutaneous closure group (S) and non-subcutaneous closure group (N) with 124 members each. Males were higher in both groups, with 65% and 61.3% in the N and S groups respectively. The majority of the patients were in the age group 50-60. The study population's mean age was 51.13 ± 15.9 years; group N had a greater mean age of 51.5 ± 15.9 years than group S, which had a mean age of 50.76 ± 16.0 years. The details of age distribution are given in Table 1.

**Table 1:** Age distribution Group S: subcutaneous closure group; group N: non-subcutaneous closure group

Age groups (in years)	Group N (n)	(%)	Group S (n)	(%)
21 to 30 years	13	10.6	15	12.1
31 to 40 years	17	13.8	19	15.3
41 to 50 years	23	18.7	22	17.7
51 to 60 years	33	26.8	31	25.0
61 to 70 years	24	19.5	21	16.9
71 to 80 years	10	8.1	16	12.9
81 to 90 years	4	2.4	0	0.0

Superficial SSI was higher in the group without subcutaneous tissue closure, with a percentage of 14.5 where as the S group had 10.5% superficial SSI rate. Chi-square test value 0.922; d.f 1; p-value 0.337. The numbers are given in Table 2.

**Table 2:** Superficial incision infection among the two groups Group S: subcutaneous closure group; group N: non-subcutaneous closure group

Superficial incision infection	Group N (n)	(%)	Group S (n)	(%)
Absent	106	85.5	111	89.5
Present	18	14.5	13	10.5

The seroma of the wound showed a higher number of patients with seroma in group N and less seroma in group S. This difference was statistically significant with a p-value < 0.05. It is depicted in Table 3.

**Table 3:** Seroma in the wound among the two groups Group S: subcutaneous closure group; group N: non-subcutaneous closure group

Seroma in the wound	Group N (n)	(%)	Group S (n)	(%)
Absent	112	90.3	119	96.0
Present	12	9.7	5	4.0

Hematoma of the wound

The hematoma of the wound was present in 13 patients in group N (10.5%) and in group S it was lower and present in six patients (4.8%). This difference was statistically significant with a p-value < 0.05.

Suture sinus formation

The presence of suture sinus formation among groups N and S showed a similar number at six patients (4.8%) each. There was no statistical significance among the two groups.

Partial wound dehiscence

The partial wound dehiscence among both groups was as follows: group S had eight patients (6.5%), and group N had seven patients (5.6%). There was no statistical significance among the two groups.

Total wound dehiscence

The total wound dehiscence showed group N with four patients (3.2%), whereas there were no patients with total wound dehiscence in group S. This difference was statistically significant.

Total duration of stay in hospital

The total duration of hospital stay showed that groups N and S had similar hospital stay duration, with nearly half in both groups in one to five days durations. There was no statistically significant difference among the groups.

**Figure 1:**
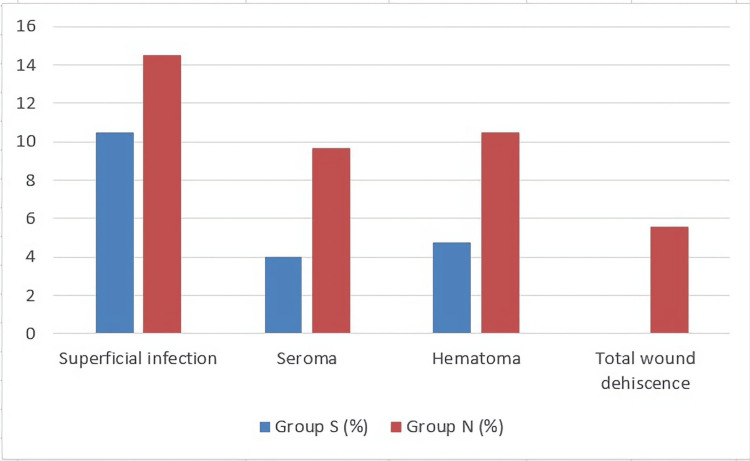
Wound complications which showed significant reductions after subcutaneous tissue closure Group S: subcutaneous closure group; group N: non-subcutaneous closure group

The final rate of all studied complications with significant differences in both groups is summarized in Figure 1.

**Figure 2:**
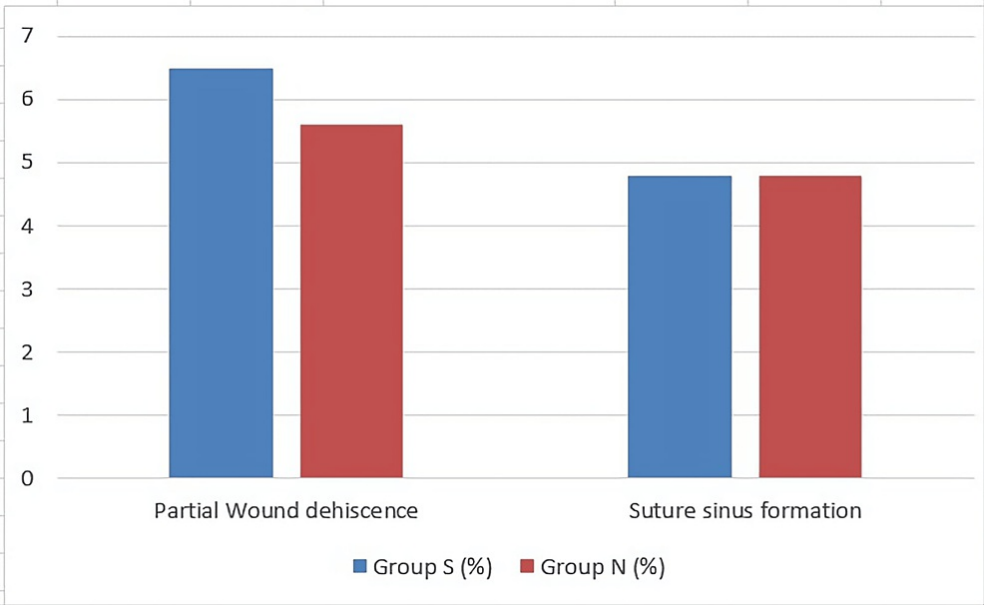
Wound complications with similar rates in both groups Group S: subcutaneous closure group; group N: non-subcutaneous closure group

The wound complications which were similar in both study groups are summarized in Figure 2.

## Discussion

The subcutaneous closure group (group S) and the non-subcutaneous closure group (group N) were the two groups randomly assigned to participate in our study. Each of the two groups randomly included a total of 124 patients. The study had 248 patients in total. The gender distribution revealed a higher proportion of males in both groups. The study population's mean age is 51.13 ± 15.9 years, which is comparable to the studies of Paral et al. [9] and Bawa et al. [10].

In our investigation, group N exhibited a higher percentage of superficial incision infections (14.5%), a considerably larger number of patients with wound seromas (9.7%), and a significantly higher number of wound hematomas (10.5%) in 13 patients in group N. Groups N and S displayed comparable numbers of suture sinus developments, and both groups displayed comparable partial wound dehiscence. Group N had a considerably higher number of total wound dehiscence. Groups N and S had similar hospital stays, according to the overall length of stay displayed. The only outcomes that subcutaneous suturing significantly improved were infection, seroma, and total wound dehiscence; subcutaneous and non-subcutaneous sutures did not differ significantly in the remaining categories. According to the reviewed study by Paral et al. [9], half of the patients in each group had subcutaneous suturing of the subcutaneous fat tissue. There were no statistically significant differences in the percentages of infectious and non-infectious wound complications between the groups with and without subcutaneous suturing. The study's findings indicate that skipping subcutaneous adipose tissue suturing does not alter the risk of either infectious or non-infectious wound complications. Until the third post-operative day, we observed partial wound dehiscence in 1.8% and non-suture groups, respectively, but no complete wound dehiscence cases.

Similarly, clean-contaminated surgery with subcutaneous suturing (group A) and clean-contaminated surgery without subcutaneous suturing (group B) were the two primary categories into which Bawa et al. [10] separated the patients. Regarding the complications of infectious and non-infectious wounds, there were no statistically significant differences. These findings imply that the incidence of infected or noninfectious wound complications is not increased by forgoing subcutaneous adipose tissue suturing. In agreement with our research, Islam and Ehsan [11] discovered that group I's surgery took an average of 7.5 minutes less time than group II's.

Patients in group I expressed satisfaction that their stitches remained intact and expressed satisfaction with the outcome of the procedure. The results of this study showed that although there was no difference between groups I and II in terms of scar tissue formation or wound infection rates, group I had shorter recovery times and happier patients. Husslein et al.'s investigation [12], which corroborated ours, discovered that the non-closure group had noticeably more hematomas (25%) than the closure group (4%).

Between groups, there was no difference in the length of operation, SSI, seroma development, or wound disruption. This study concluded that suture closure of the subcutaneous fat at CS has no effect on long-term cosmetic results. Similar findings to our investigation were reported by Patel et al. [13]. According to a meta-analysis conducted by Chelmow et al. [14], suturing the subcutaneous adipose layer reduced the likelihood of wound disruption because it prevented the formation of seroma. Around 7.7% and 11.5% of the suture and non-suture groups, respectively, exhibited seroma collection, according to Allaire et al. [15]. The variations, however, lacked statistical significance. Infections occurred in 4% of each group's cases. According to research by Kong et al. [16], there were no appreciable variations in the frequency of wound infection.

Post-operative complications are dependent on many other factors including patients' comorbidities, operation theatre factors, adequate patient preparation, etc. The main limitation of this study is these confounding factors that must have contributed to the final outcome.

## Conclusions

Subcutaneous tissue suturing of laparotomy wounds in elective surgeries would be helpful in reducing the rate of superficial SSI, seroma, hematoma and total wound dehiscence. Other wound complications such as partial wound dehiscence and suture sinus formation were not altered by suturing the subcutaneous layer. It did not help in reducing the duration of hospital stay post-operatively.
